# The Use of Total Thrombus Formation Analysis System as a Tool to Assess Platelet Function in Bleeding and Thrombosis Risk—A Systematic Review

**DOI:** 10.3390/ijms22168605

**Published:** 2021-08-10

**Authors:** Joanna Sikora, Aleksandra Karczmarska-Wódzka, Joanna Bugieda, Przemysław Sobczak

**Affiliations:** 1 Research and Education Unit for Experimental Biotechnology, Department of Transplantology and General Surgery, Faculty of Medicine, Collegium Medicum in Bydgoszcz, Nicolaus Copernicus University in Toruń, 85-094 Bydgoszcz, Poland; akar@cm.umk.pl (A.K.-W.); joanna.bugieda@cm.umk.pl (J.B.); 2 Department of Hematology, Collegium Medicum in Bydgoszcz, Nicolaus Copernicus University in Toruń, 85-094 Bydgoszcz, Poland; przemyslawsobczak02@gmail.com

**Keywords:** T-TAS, platelet function, bleeding, thrombosis, blood coagulation

## Abstract

Background. Today there are many devices that can be used to study blood clotting disorders by identifying abnormalities in blood platelets. The Total Thrombus Formation Analysis System is an automated microchip flow chamber system that is used for the quantitative analysis of clot formation under blood flow conditions. For several years, researchers have been using a tool to analyse various clinical situations of patients to identify the properties and biochemical processes occurring within platelets and their microenvironment. Methods. An investigation of recent published literature was conducted based on PRISMA. This review includes 52 science papers directly related to the use of the Total Clot Formation Analysis System in relation to bleeding, surgery, platelet function assessment, anticoagulation monitoring, von Willebrand factor and others. Conclusion. Most available studies indicate that The Total Thrombus Formation Analysis System may be useful in diagnostic issues, with devices used to monitor therapy or as a significant tool for predicting bleeding events. However, T-TAS not that has the potential for diagnostic indications, but allows the direct observation of the flow and the interactions between blood cells, including the intensity and dynamics of clot formation. The device is expected to be of significant value for basic research to observe the interactions and changes within platelets and their microenvironment.

## 1. Introduction

Haemostasis is an important process that maintains the integrity of the circulatory system and minimises blood loss upon vascular damage. When a blood vessel-wall is injured a number of concomitant events occur. Initially, circulating platelets are recruited to the site of the injury where they are activated to become a platelet plug. Secondly, blood coagulation is triggered by tissue factors resulting in thrombin generation and fibrin formation. Fibrin monomers are then cross-linked into insoluble strands that serve to stabilize the loose platelet clot. The formation of fibrin and the platelet plug become major components of the developing thrombus at the sight of injury to prevent bleeding [1]. Hemostasis is a complex process that is contingent on the complex interaction of platelets, plasma coagulation cascades, fibrinolytic proteins, blood vasculatures and cytokine mediators, which promote the inflammatory reaction and the accumulation of cells required for wound repair after injury [2]. A number of instruments have now become available to investigate bleeding disorders by assessing platelet function defects.

The Total Thrombus Formation Analysis System (T-TAS) is an automated microchip flow chamber system for the quantitative analysis of the thrombus formation process under blood flow conditions. Under arterial flow conditions, white thrombi consisting of numerous activated platelets and fibrin fibers are formed, whereas red thrombi forming in the venous circulation predominantly consist of fibrin and red blood cells [1]. When introducing flow chambers into clinics, the type of flow chamber, coating of surfaces, collection and storage of blood before start of analysis, recording of digital images and the method of image quantification should be addressed. The device, therefore, offers the potential advantage of simultaneous assessment of both platelet and coagulation defects.

Using T-TAS for research, the white thrombus formation occurring in the chip can be observed and quantitatively analysed under arterial and venous flow conditions. Moreover, it observes the time course of thrombus formation in whole blood flowing in a simulated blood vessel at a constant rate. White thrombus growth rate and stability are easily evaluated through pressure waveforms and indicators. In addition, real time visual observation of thrombus formation with a camera, the small amount of whole blood per assay (320 to 450 μL) and rapid measurements (10 to 30 min) are important features. There are two types of chips, the PL-chip and AR-chip.

**Figure 1:**
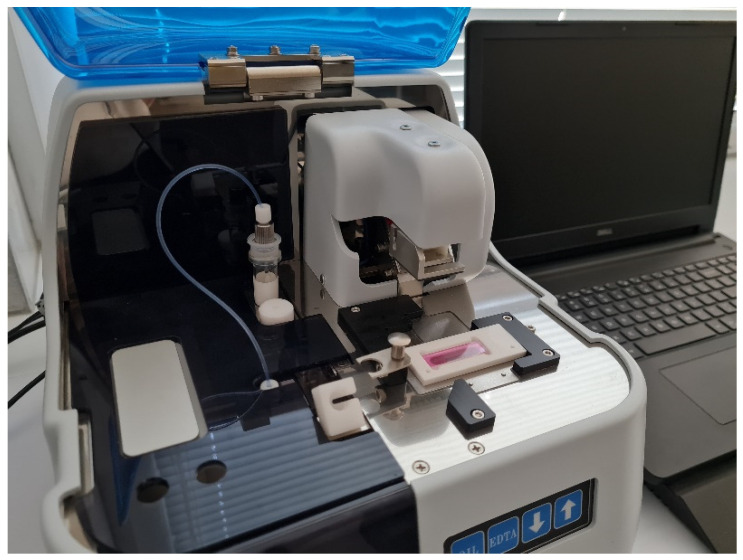
Total thrombus formation analysis system.

The T-TAS is a device comprised of a tabletop instrument controlled by a dedicated PC and a disposable, single-use flow chamber (Figure 1). The PL-chip is designed to specifically measure platelet thrombus formation (PTF) under physiological conditions on a collagen-coated analytical pathway consisting of microcapillary channels. PTF is a direct indicator of the patient’s primary hemostatic function. The assay is performed under arterial flow conditions using an anticoagulant that inhibits thrombin and factor Xa, blocking the coagulation cascade and allowing the PL-chip to specifically measure only PTF. During the assay, the blood sample is exposed to arterial shear stresses in the presence of a collagen-coated surface, which causes platelet attachment to collagen mediated by the von Willebrand factor (vWF), and platelet activation. The vWF represents a high-molecular-weight adhesive glycoprotein that plays an essential role in primary hemostasis by promoting platelet adhesion to the subendothelium and platelet plug formation at the sites of vascular injury [3]. Platelet activation causes the release of endogenous factors contained within the platelets that recruit and activate other platelets and cause aggregation and platelet thrombus formation. The growing platelet thrombus causes occlusion of the microcapillary channels, which increases the flow pressure within the assay chip. The process of platelet thrombus formation in the flow chamber is continuously monitored by a pressure sensor that tracks pressure changes in the flow path.

The AR-chip has a built-in simulated blood vessel in which the inner lumen is coated with collagen and tissue factor. After adding Ca^2+^ in the simulated vessel, citrated whole blood is activated by the collagen and tissue factor. Then, a very firm thrombus is formed by activated platelets and coagulation factors. Therefore, the AR-chip enables assessment of the cooperative capacity of platelets and the coagulation system in thrombus formation. Since the pressure curve reflects the rate of thrombus formation and thrombus firmness, coagulability and platelet function can be assessed by reading the pressure curve [4]. T-TAS, unlike other techniques that usually assess either platelet activity or the clotting system, allows both components to be assessed simultaneously. The device, by carrying out the process in ex vivo conditions, allows the observation of the components of the coagulation system as in physical conditions.

The flow chamber technique has been popularized during the last years with the introduction of commercial flow chambers, also custom adapted, such as Ibidi, Venaflux, Bioflux, and Glycotech. The Scientific and Standardization Committee (SSC) of the International Society on Thrombosis and Haemostasis (ISTH) has recently reviewed this field. The majority of laboratories use collagen coatings, but also esculetin, fibrinogen, vWF, and other synthetic peptides. In this article, we review the use of T-TAS in various fields of medicine, from classical use in cardiology to new reports of its use in diseases such as polycystic ovary syndrome and COVID-19.

## 2. Methods

**Figure 2:**
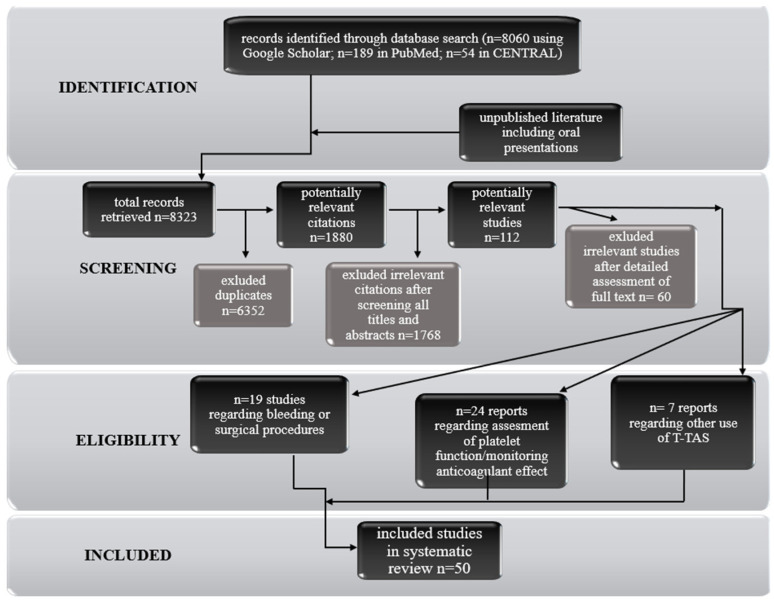
Flow chart of studies selection.

An investigation of recent published literature was conducted based on PRISMA. Briefly, a database search (date of search 10 May 2021) including PubMed, CENTRAL and Google Scholar databases. The following keywords were applied: “T-TAS”, “Total thrombus formation analysis system”, “microchip flow chamber system”. References of retrieved studies were searched manually for additional studies and reviews. Reviews were also considered by sources of citations of relevant studies and interpretation of their results. Duplicate and multiple citations and reviews not containing any relevant information were excluded. Eventually, 50 original reports directly related to the use of the total thrombus formation analysis system were considered eligible for inclusion in the review. The articles were divided into the categories of bleeding, surgical procedures, assessment of platelet function, monitoring anticoagulant effect, vWF, and others. A detailed flow chart of the studies selection is shown in the Figure 2.

## 3. Results and Discussion

### 3.1. Assessment of Platelet Function

This section presents 12 studies, two of which (17%) related to diabetes. In addition, the effects of anticoagulants (42%) and studies of healthy people were investigated. For the presented studies, the PL-chip was used predominantly (42%), while the use of the AR-chip or both chips were used less frequently (33% each). All results from the studies are presented in Table 1.

Typically, evaluation of platelet function is performed by methods related to aggregometry; however, a new approach is the analysis of thrombus formation on microchips [5]. Minami et al. [6] described the investigation of platelet thrombus formation (PTF) at high shear rates using T-TAS as a screening test for diagnosis in patients with platelet storage pool disease (SPD). Platelet counts and sizes were demonstrated, broadly similar to normal controls, by using routine coagulation tests such as the PRP313 M instrument Multiplate Analyzer. Evaluation of platelet thrombus formation was performed with the PL-chip. Authors suggested that T-TAS offers considerable potential as a screening test for functional abnormal platelet aggregation in SPD [6].

In 2021 Ghirardello et al. [7] undertook a study of COVID-19, in which researchers watched thrombus formation in SARS-CoV-2 patients using T-TAS. Platelets were assessed using the PL-chip at different stages of the disease. Scientists observed that parameters such as the area under the flow-pressure curve (AUC) and occlusion time (OT) were significantly different in COVID-19 patients (regardless of how severe the disease was) compared to healthy controls. The study showed that in COVID-19 patients, platelet thrombus formation assessed by T-TAS was impaired during the first week after hospital admission compared to controls and patients at later disease stages. In contrast, there was no observed difference in PTF among patients with different illness severity, although all patients, independently from the intensity care level, showed reduced platelet thrombogenicity compared to controls. The platelet chip-based assay embedded in T-TAS explores the three major steps in PTF: platelet adhesion mediated by vWF, the release of endogenous platelet agonists, and platelet aggregation [7]. Authors hypothesized that platelets exert an immunological role during the first stage of COVID-19 at the expense of their haemostatic functions. Sustained platelet stimulation and degranulation during the onset of virus replication may downregulate platelet haemostatic function and stimulate platelet immune response [8].

Tsujii et al. [9] analysed clot formation in patients with Kawasaki disease (KD). The study consisted of the assessment of PTF under various flow conditions (using a PL chip). The aim of the study was to determine the platelet activation process and to evaluate the effect of aspirin antiplatelet therapy in patients with acute KD. Researchers observed that the acute KD phase has an early onset and shows low PTF stability, but it wasn’t specific. Aspirin suppressed platelet activation regardless of dose and duration of drug use [9].

The topic of anticoagulants was dealt with by Idemoto et al. [10] in which scientists assessed the effect of antithrombotic abilities of various novel oral anticoagulants (NOACs) using T-TAS as a useful tool for monitoring antithrombotic effects using both types of chips, and performed common coagulation tests. Authors evaluated the area under the flow pressure curve to quantify antithrombotic ability using T-TAS. As a result, they showed significant decreases in AR-AUC and PL-AUC with all anticoagulants except the rivaroxaban group [10].

Ożegowska et al. [11] analysed T-TAS in terms of determining the risk of coagulation disorders in women with polycystic ovary syndrome (PCOS). This study compared T-TAS (AR-chip) with classic coagulation parameters such as RBC, white blood cells (WBC), haematocrit, platelets, antithrombin, d-dimer, fibrinogen, prothrombin time (PT), international normalized ratio (INR) and prothrombin index (PI). Researchers observed that the area under the curve (AUC30) had higher values in PCOS patients compared to controls. The T-TAS parameters were also independent of body mass index (BMI). Researchers found that increased triglyceride levels in PCOS patients increased AUC30 and decreased the time of thrombus formation initiation (T10) and OT, but more research is needed to determine the usefulness of T-TAS in this group of patients [11]. On the other hand, Osiński et al. [12] assessed whether in pregnant women with type 1 diabetes glycaemic control influences the prothrombotic and antifibrinolytic status. T-TAS (AR-chip) was used for the analysis to check if it is more sensitive than standard methods. Researchers observed a relationship between glycated haemoglobin (HbA1c) levels and T10 values. It has been proven that poor glycaemic control in pregnant women with diabetes is correlated with greater thrombogenicity. The results indicated that T-TAS is better for the diagnosis of the prothrombotic state than, for example, activated partial thromboplastin time (APPT) or PT. Mean platelet volume (MPV) and d-dimer measurements can also be used to predict thrombotic status [12]. The blood glucose test was also undertaken by Yamamoto et al. [13]. In addition to glycaemic control, they also analysed hypoglycaemia using T-TAS and, more precisely, its effect on clot formation. Results of the study proved that glycaemic control reduces thrombogenicity for a short time. Then, a decrease in blood glucose was observed which increased the activity of platelets, what is associated with an increase of adrenaline. Therefore, scientists suggest that glycaemic control is important in critical situations and that it is relevant to avoid hypoglycaemia [13]. In diabetes, platelet dysfunction occurs due to intracellular hyperglycaemia related to insulin-independent glucose transporters in the platelet cell membrane. In DM patients it is observed that there is an increase in GpIIb/IIIa expression and amplification of ADP/P2Y12 signaling, and there is also an increase in the production of reactive oxygen species (ROS), which contribute to platelet dysfunction [14].

Hosokawa et al. [15] assessed the variability of PTF in patients with heart disease. The relationship between residual PTF and agonist-induced platelet aggregation or flow cytometry (FCM) parameters such as phorbol myristate acetate (PMA) and P-selectin (CD62P), was determined. Therefore, T-TAS (PL-chip), a Multiplate Analyzer and flow cytometry were used for analysis of patients receiving antiplatelet therapy (aspirin or aspirin and clopidogrel). The results obtained by the investigators indicated the effect of dual antiplatelet therapy (DAPT) on the T-TAS AUC10 parameter values reflecting platelet thrombus persistence, and were correlated with the inhibition of P2Y12 at arterial shear rates. Measurements from the Multiplate showed that during DAPT clopidogrel inhibited PTF more than aspirin. The authors suggest that the assessment of PTFs using T-TAS along with conventional platelet function tests will enable the analysis of residual thrombogenicity as well as the optimization of antiplatelet therapy [15].

In the context of the assessment of thrombogenicity, Yamaguchi et al. [16] analysed the blood of healthy people. The results from the T-TAS (PL and AR-chip) were compared with other methods for assessing platelet function such as light transmission aggregometry (LTA), VerifyNow (P2Y12) and the platelet function analyser PFA-100. Scientists observed that the obtained measurements can reflect the individual properties of PTF. PL-chips were more sensitive than in the case of the PFA-100. However, there was no correlation in the LTA and VerifyNow results with T-TAS. Scientists suggest that T-TAS may be useful for monitoring the prevention of thrombotic diseases [16].

In contrast, the research of Taune et al. suggests [17] that T-TAS may not be entirely effective in all cases. Their research showed that rotational thromboelastometry clotting time (ROTEM-CT) might prove more effective for the evaluation of the concentration of dabigatran and antithrombotic effects. The Rotational thromboelastometry (ROTEM) variable clotting time using Ex-tem and Fib-tem triggers correlates to a high degree (*r*-value of around 0.9) with the plasma dabigatran concentration throughout the spectra of concentrations investigated in this study. Further, T-TAS was able to detect differences in haemostasis under flowing conditions during the dosing interval of dabigatran in a real-life population of patients treated with dabigatran. However, the correlations between T-TAS variables and actual dabigatran plasma concentrations were weaker than those observed for ROTEM-CT [17].

Another T-TAS study in the context of thrombogenicity assessment was the analysis by Hosokawa et al. [18]. Scientists assessed the effect of the type 1 plasminogen activator inhibitor (PAI-1) in platelets on thrombogenicity. The AR-chip was used to test samples from healthy humans as well as from wild-type and PAI-1-deficient mice. Researchers found that blood flow and clot components influenced the thrombolytic efficiency of t-PA, and that thrombolysis was regulated by PAI-1 released from platelets. The authors suggest that the combination of t-PA with arterial shear-flow antiplatelet drugs will enable the evaluation of the effectiveness of t-PA treatment, and at the same time improve thrombolytic therapies in arterial circulation [18]. Miike et al. [19] investigated the effect of high doses of the main thrombin inhibitor antithrombin on the blood coagulation process and platelet function. In this study Point-of-Care Testing (POCT), such as T-TAS (AR and PL-chip) and ROTEM (NATEM) were used to assess changes in coagulation in healthy people. The results obtained from the analysis with the AR-chip indicated that antithrombin has an antithrombotic effect which depends on the dose administered and that the use of a heparinoid is not required for this. In contrast, PL-chip measurements showed a dose-dependent effect on clot formation in a suppressive manner [19].

### 3.2. Monitoring the Anticoagulant Effects

This section summarizes 12 studies involving different anticoagulant therapies. Most (50%) of the studies presented concerned anticoagulants such as rivaroxaban, apixaban, dabigatran, warfarin and abciximab. In addition, antiplatelet drugs (33%) such as aspirin and clopidogrel were used in the studies. Analysis was conducted with the highest percentage of patients with atrial fibrillation (AF) (25%). Only healthy people participated in five studies (42%). The least common in the presented studies were the analysis of patients with cerebrovascular disease and CAD (8% and 17%, respectively). Researchers using T-TAS mostly selected both available chips for analysis (AR-chip and PL-chip) or decided to use only the AR chip (42%). The PL chip was used in three studies (25%). Patients with various cardiovascular diseases often require anticoagulation therapy, and evaluation of the effectiveness of anticoagulants should be monitored for this type of therapy. T-TAS is one a device used to monitor therapy with anticoagulants [1]. Comparison of articles on the topic of monitoring the anticoagulant effects is presented in Table 2.

Hosokawa et al. [20] presented the T-TAS system for the quantification of white thrombus formation (WTF). They also compared the effectiveness of different anticoagulants under different flow conditions. Several diagnostic devices have been recently developed for the evaluation of pathogenesis and therapies related to thrombotic and haemorrhagic disorders. The authors suggested that this device could be useful for the evaluation of different types of anticoagulant therapies [20]. In an in vitro and ex vivo study, Sugihara et al. [21] evaluated the usefulness of the T-TAS for monitoring the anticoagulant effects of rivaroxaban or apixaban. The first part of the study involved an in vitro experiment. The blood samples were obtained from all patients at two time points: the first time point before drug administration and the second time point 4 h after drug administration. Next, all blood samples were evaluated using the T-TAS. In vitro studies found significant differences in AR-AUC values between blood samples with rivaroxaban and apixaban and the control group. Similar dependencies were obtained in the study ex vivo. At the first time point, the AR-AUC value was significantly higher compared to the second time point. This study suggests that a T-TAS with an AR-chip was a useful tool for monitoring the anticoagulant effects of rivaroxaban and apixaban, and may provide accurate quantitative results [21].

On the other hand, Yamazaki et al. [22] showed that platelet thrombus formation measurement using the T-TAS system was useful for evaluating the efficacy of treatment with aspirin and clopidogrel. These drugs were given to patients both alone and in combination. Despite the numerous methods and instruments (LTA, VerifyNow, flow cytometry) for measuring platelet function, the correlation among these tests was relatively low. The differences between these assays may also be attributable to the fundamental measurement principle of each test. These findings suggest that T-TAS in combination with LTA or the VerifyNow system may be useful for evaluating the efficacy of antiplatelet therapy by several agents, as both the specific effects of each agent and the total residual thrombogenicity can be measured using this approach. In addition, the present results show that elevated residual platelet thrombogenicity in patients treated with clopidogrel alone is associated with the severity of carotid or intracranial arterial stenosis, which are both reported to be risk factors for ischemic cerebrovascular events. Therefore, the combined analyses of PTF with LTA or the VerifyNow system would potentially provide useful information for evaluating the risk-to-benefit balance of antiplatelet therapies [22].

Arima et al. [23] evaluated in a cross-sectional study the utility of T-TAS in the assessment of the effects of different antiplatelet therapies in patients with coronary artery disease (CAD). T-TAS measured platelet thrombus formation AUC at various settings, i.e., 1500 s^−1^ (PL18-AUC10) and 2000 s^−1^ (PL24-AUC10) for the PL-chip, and 300 s^−1^ (AR10-AUC30) for the AR-chip. Obtained results correlated with the VerifyNow P2Y12 assay. Results suggest that the PL24-AUC10 parameter may be useful for evaluation of antiplatelet therapy in CAD patients [23]. Zheng et al. [24] evaluated T-TAS for the measurement of clot formation under flow conditions in patients with CAD undergoing percutaneous coronary intervention (PCI) treated with DAPT. DAPT included aspirin and either clopidogrel, prasugrel or ticagrelor. In addition to T-TAS, this study also used a Multiplate to be able to determine the high platelet reactivity in patients receiving clopidogrel, and thus justify switching to prasugrel. On the basis of the obtained results, the researchers suggested that in patients treated with antiphlogistic therapy using the PL-chip in the T-TAS, an overall assessment of the primary haemostatic capacity is possible [24].

Hosokawa et al. [25] evaluated antithrombotic efficacies of dabigatran and rivaroxaban in combination with antiplatelet agent (aspirin and AR-C66096) or without an antiplatelet agent in vitro by T-TAS. Furthermore, investigators assessed the impact of thromboplastin levels on the antithrombotic effects of these drugs using two types of microchips, i.e., high thromboplastin (HTP; 100 µg mL^−1^) and low thromboplastin (LTP; 25 µg mL^−1^). The results obtained by Hosokawa et al. indicate that dabigatran in concentrations 500 and 1000 nM substantially inhibited the thrombus formation process at both shear rates regardless of the tissue thromboplastin levels, while rivaroxaban at a concentration of 1000 nM delayed the thrombus formation process only. Investigators found the anticoagulant efficacy of dabigatran and rivaroxaban evaluated under flow conditions markedly differed in comparison to static conditions [25].

Based on one patient, Norimatsu et al. [26] assessed circadian variation in the anticoagulant effect of rivaroxaban by T-TAS. This study measured the effects of rivaroxaban in a blood sample from a patient with paroxysmal atrial fibrillation at six time points (before and 2, 4, 8, 11 and 23 h after the oral administration of rivaroxaban). This trial used two types of microchips (AR-chip and PL-chip) at flow rates of 4 μg/mL and 18 μg/mL, respectively. In the study patient, administration of rivaroxaban did not require another dose until the next morning. This study opened the way for the evaluation of patients with paroxysmal AF (PAF) using T-TAS on a wider scale [26].

On the other hand, Ishii et al. [27] determined differences in the anticoagulation patterns of warfarin and DOACs in patients with AF who had undergone radiofrequency catheter ablation. The total thrombus formation analysis system is useful for monitoring the anticoagulant effects of DOACs and predicting periprocedural bleeding events. The AR-AUC correlated weakly and negatively with plasma concentrations of DOACs, and the levels at on-anticoagulant were lower in all groups than at off-anticoagulant. AR-AUC levels at off and on-anticoagulant were identical among four patient groups (warfarin group, dabigatran group, rivaroxaban group and apixaban group) [27]. 

Clifford et al. [28] looked at treatment with aspirin only or ticagrelor, DAPT consisting of aspirin and ticagrelor, rivaroxaban only, and the so-called dual pathway (DP) therapy consisting of aspirin and rivaroxaban. Both chips in T-TAS were used for analysis. From the results, the researchers observed that the composition DP showed a better anticoagulant effect, which may be due to an additive anticoagulant mechanism. The use of the PL-chip made it possible to demonstrate that, compared to acetylsalicylic acid, DP does not provide evidence suggesting a greater antiplatelet effect. From the data obtained, it was possible to conclude that the best treatment regimen to prevent blood clots is DAPT. Summarizing their research, the authors suggest that DP guarantees antithrombotic synergism with the help of anticoagulant action (without isolated antiplatelet synergism) [28].

Skalski et al. [29] investigated substances isolated from a sea buckthornin the context of antiplatelet properties in whole blood. The authors suggested that the primarily fraction A, which acts as an anticoagulant, acts in a different way than through the P2Y12 receptor [29]. Lis et al. [30] studied the antiplatelet effect of various fractions isolated from different parts of the plant in whole blood. This potential was analysed using flow cytometry (including the VASP test), T-TAS (PL-chip) and electrophoresis (analysis of proteome changes). The results obtained from the PL-chip indicated that only the fraction derived from dandelion leaves (named C) shows anticoagulant properties. Reduction in the expression of P-selectin and glycoprotein IIb/IIIa (active form) was observed in both phenolic acid-rich fractions (named C and D). On the basis of the results, the authors suggested that in order to prevent and treat cardiovascular diseases related to excessive activation of blood platelets, a plant such as a dandelion can be introduced into the diet in the form of a leaf salad or flower syrup [30].

A novel approach in the context of antithrombotic therapy is the development of a vaccine with a long-lasting antithrombotic effect. Shimamura et al. [31] analysed the immunogenic S100A9 epitope in monkeys as well as the anticoagulant effect of the antibodies produced. In addition, it was determined if the said antibodies specifically recognized the epitope. For these purposes, inter alia, T-TAS (PL-chip), enzyme immunoassay and Western blot analysis were used. The peptide vaccine designed by the researchers (against S100A9) effectively inhibited the clot formation process in the blood of monkeys. The anticoagulant effect was not the same in the whole study group. The authors, despite the need to determine the optimal dose, time of effective action and safety, suggest that such a vaccine may prevent recurrence of a stroke caused by the patient’s failure to follow the doctor’s instructions [31].

### 3.3. Bleeding

This section compiles 14 studies, with the highest percentage of studies in patients taking anticoagulants (36%), patients with vWD (21%) with CAD (29%) and after PCI (29%). Tests were performed less frequently include studies involving human and mouse populations (14%) and patients requiring platelet transfusion (7%). Both PL-chip and AR-chip were mostly used (approx. 64%). 

Ogawa et al. [32] conducted research regarding the influence of blood flow on blood thrombus formation in patients with haemophilia. Haemophilia is a haemorrhagic diathesis in which two types are distinguished: types A and B. Both types are inherited from parents’ coagulation disorders associated with the generation of insufficient amount of thrombin, which should prevent bleeding. In type A, the reason is factor VIII deficiency, while in type B it is caused by factor IX deficiency. Patients are at risk of recurrent bleeding, which can be life-threatening [33]. Research was conducted on using mice models and humans, which showed that the fragility of the thrombus in haemophilia correlates with shear rates. To identify the pathogenesis of bleeding researchers focused on thrombus formation in whole blood. It is worth to noting that evaluation of platelet-mediated haemostatic function has been difficult because platelet function is generally tested in anticoagulated blood, and coagulation tests are performed in platelet-poor plasma. Moreover, these trials were conducted in haemophilia patients under static conditions. The results showed that dense networks of fibrin fibres surrounding platelets were visible in normal blood, whereas coarse fibrin structures with large pores were observed in FIX-inhibited blood. Further investigations are warranted to evaluate clinical utility of this microchip-based flow chamber system for haemostatic management in haemophilia [33]. 

Nakajima et al. undertook research on the use of T-TAS to analyse clinical severity in patients with type N2 vWD. There are three main types of vWD (additionally, type 2 is divided into four subtypes: A, B, M and N). Type 2N in vWD is characterized by low plasma levels of factor VIII and the damage in the interaction between factor VIII and vWF. Consequently, it resembles haemophilia [1]. Both chips (AR and PL) were used in the study. The results indicated that the AR-chip can be used to determine the ability of bleeding and the effect of treatment on the patient, which is the infusion concentrates of FVIII/vWF complex. In order to improve the laboratory analysis of patients with type 2N von Willebrand disease, a cohort study of more patients should be conducted [34].

Apart from the above studies, there are few other publications about bleeding. Yaoi et al. [35] demonstrated that the severity of anaemia is directly proportional to uncontrolled bleeding. In this study haemostasis was investigated using a novel microchip flow-chamber system in an anaemia patient with von Willebrand disease. The effects of red blood cells in the total thrombus analysis system were examined using reconstituted whole blood at various haematocrit levels. However, transfusions of RBCs-resolved bleeding symptoms assessed by T-TAS at 1000 s^−1^. The study results showed that red blood cells play an essential role in primary haemostasis under high shear conditions in the case of resistance to bleeding in patients with anaemia. Further studies, to determine the wider utility of the technique for monitoring anaemic patients should be conducted. Thus, T-TAS identifies disordered haemostasis in the presence of anaemia [35]. In contrast, Takashio et al. [36] analysed use of T-TAS to detect acquired von Willebrand syndrome (AVWS) in patients with a left ventricular continuous flow assistive device. Both types of chips were used for this study. It was found that a high level of the AR10-AUC 30 parameter may indicate a high risk of thrombosis. On the other hand, a low level of this parameter may be useful in determining the risk of bleeding [36]. Nakajima et al. [37] studied detection of congenital disorders of primary haemostasis using T-TAS. Analysis was performed in patients with von Willebrand disease types 2A, 2B and type 3, as well as with platelet dysfunction such as Bernard-Soulier syndrome and Glanzmann’s thrombasthenia. In addition to T-TAS, a Multiplate Analyzer was used to determine platelet function. PL and AR-chips were used in a screening test to determine the sensitivity of T-TAS. Researchers found that the T-TAS PL-chip enabled the precise determination of inherited primary haemostatic disorders. The results of the study proved that the Multiplate can also be used for such screening. Despite the need for further research, scientists concluded that T-TAS (PL-chip) can be clinically used as a rapid screening test for congenital haemorrhages [37].

The research of Oimatsu et al. [38] investigated the association between T-TAS measurements and periprocedural bleeding. In a study conducted among 313 patients with CAD who underwent PCI by the femoral approach, they discovered that a low PL-AUC level was an indicator of the potential of major bleeding events [38]. However, Ito et al. [39] in their original research proved the usefulness of T-TAS as a novel, quantitative assessment of whole-blood thrombogenicity, and showed that T-TAS as a useful marker for prediction of periprocedural bleeding events in patients undergoing catheter ablation for atrial fibrillation. Furthermore, there was no difference between novel oral anticoagulants (NOACs) and the warfarin group except for a higher rate of patients with a HASBLED score of zero and use of antiarrhythmic drugs in the NOAC group. There were no significant differences in baseline AR-AUC, PL-AUC, PT-INR and APTT levels among all NOACs groups (dabigatran group, rivaroxaban, and apixaban group), except for a higher APTT level in the dabigatran group compared with the rivaroxaban group. What is clinically important is that periprocedural bleeding events were observed in 16.4% of the atrial fibrillation patients undergoing catheter ablation. The authors observed that AR-AUC level measured by T-TAS was an independent and significant tool for assessment of the efficacy of warfarin and NOACs in AF patients who underwent catheter ablation and could be a useful predictor of periprocedural bleeding events in such patients [40]. 

Nakanishi et al. [41] conducted similar studies to determine the risk of bleeding in patients undergoing PCI. The Academic Research Consortium for High Bleeding Risk (ARC-HBR) criteria were used in this study. It was observed that thrombogenicity of ARC-HBR positive patients was lower. Combining ARC-HBR with AR10-AUC30, together with statistical methods predicted the risk of bleeding after PCI over a period of 1 year. Consequently, measuring AR10-AUC30 before PCI may be useful in determining thrombogenicity and bleeding risk after surgery, but further studies in a larger population are needed [41]. The same researchers undertook the study of the relationship between thrombogenicity and haemodialysis in a group of patients undergoing PCI using T-TAS. In addition to determining the relationship between haemodialysis and low thrombogenicity, the impact of haemodialysis on bleeding episodes during the 1 year was also analysed. The researchers observed that in haemodialysis patients the levels of parameters such as PL18-AUC10 and AR10-AUC30 were low. These parameters also had lower values in the presence of bleeding in patients than in the absence of bleeding. The authors suggested that T-TAS may be used to monitor the thrombogenicity of haemodialysis patients undergoing PCI to determine the risk of bleeding [39]. The prediction of annual bleeding in patients with CAD was analysed by Mitsuse et al. [42]. The study assessed the usefulness of T-TAS in predicting the annual bleeding risk in patients with CAD undergoing anticoagulant treatment. Researchers indicated that a low level of AR 10-AUC is associated with a significant risk of bleeding in patients with coronary heart disease over a year [42].

Al Ghaithi et al. [43] used T-TAS for the measurement of in vitro thrombus formation under different shear stress conditions. AR-chip thrombus formation was inhibited with rivaroxaban. T-TAS showed agreement with LTA in about 82% of patients tested so far. The onset (T10) of thrombus formation in wildtype mice was shorter when compared to human studies [43]. Results of a study by Ichikawa et al. [44] determined the effect of the therapy of many anticoagulants on bleeding complications. Patients with stable CAD were treated with antiplatelet drugs (mono or dual antiplatelet therapy) and anticoagulants (oral anticoagulants or vitamin K antagonists), and clot stability was determined using both chips. The investigators concluded that the level of AR4-AUC30 enables the prediction of bleeding complications in stable patients with CAD [45].

Ogawa et al. [46] examined the effects of haemodilution and procoagulant factors such as: von vWF, prothrombin complex concentrates on the thrombus formation process using a microchip-based flow-chamber system. Based on this study, the differences between the prothrombin complex concentrate and fresh-frozen plasma were presented. Warfarin delayed the clot formation process during flow. Restoration of normal clot formation was better with the addition of three or four factor thrombin complex concentrates than with fresh-frozen plasma. The effective action of prothrombin complex concentrate (PCC) on haemostasis may be reflected in the correct level of factor II (FII) and the high value of endogenously produced thrombin [46]. Moreover, Atari et al. [44] used a modified T-TAS to investigate thrombogenicity in patients with thrombocytopenia. The researchers concluded that the results obtained by using a new HD chip can determine the risk of bleeding and detect a return to normal haemostatic platelet transfusion. This study was the first step in determining that T-TAS may have future applications in deciding the need for a platelet transfusion [44]. A comparison of the results is presented in Table 3.

### 3.4. Surgical Procedures

This section summarizes five studies covering various surgical procedures, of which 40% were total knee arthroplasty (TKA) and a smaller percentage (20%) concerned transcatheter aortic valve implantation (TAVI) and PPCI. One study looked at a pre-surgery procedure. Most of the researchers used the AR-chip, while the use of the PL-chip, or both available chips, accounted for 20%. Operation was defined as treatment performed on the organ or tissue designed to improve health or diagnosis of the disease [47]. 

Starting with orthopaedic operations, Sueta et al. [48] assessed the utility of T-TAS for the quantitative analysis of thrombogenicity in patients treated with edoxaban. This study included 20 consecutive patients who were underwent total knee arthroplasty. All patients received edoxaban to prevent venous thromboembolism. The T-TAS measurement procedure was used with two disposable microchips, the PL-chip and the AR-chip, at two time points: the before and 7 days after operation. The results suggested that analysis of thrombogenicity using the AR10–AUC30 was a significant predictor of the efficacy of treatment with edoxaban [48]. Another orthopedic procedure study was performed by Sueta et al. [49] in a prospective single-center open-label randomized controlled clinical trial (ESCORT-TKA), which evaluated the efficacy of edoxaban in reducing the incidence of venous thromboembolism after TKA using T-TAS. They observed that decreased of AR10-AUC30 was significantly associated with the edoxaban treatment. The authors suggest that edoxaban can be used to prevent venous thromboembolism (VTE) in patients after TKA [50].

Moving on to cardiac surgery, Ishii et al. [51] assessed thrombogenic activity and thrombocytopenia after transcatheter aortic valve implantation. Thrombogenicity was assessed using the AR chip before and after transcatheter aortic valve implantation (TAVI) (2, 7 and 30 days). This study also measured thrombopoietin and multimers of vWF with high molecular weight in plasma, and both parameters achieved higher values two days after TAVI than before treatment. Using the computational fluid dynamics (CFD) model, the researchers determined the effect on wall shear stress (WSS). The obtained CFD results showed that valve stenosis using TAVI improved WSS in the aortic valve and the posterior wall of the ascending aorta. The authors found that high-molecular-weight (HMW)-vWF multimers improved more quickly after TAVI than parameters measured by T-TAS. Thrombogenic activity improves 30 days after surgery, which may explain the high risk of bleeding complications after TAVI, but more research is needed [51].

Kikuchi et al. [49] used T-TAS to assess thrombogenicity in patients with ST segment elevation myocardial infarction (STEMI) undergoing primary PCI. In addition to the T-TAS (PL-chip) analysis, the researchers used VerifyNow (P2Y12). In this study, the correlation between thrombogenicity measured by T-TAS and the size of enzymatic infarction during primary PCI was determined. The authors observed that high values of the PL18-AUC10 parameter were associated with increased enzymatic infarction and impaired reperfusion, which equates to obstruction of the coronary vessels. P2Y12 reaction unit changes measured using VerifyNow differed from the results obtained with T-TAS. Total platelet thrombogenicity can be analysed using T-TAS in patients even in the acute phase of STEMI [49].

Haemodilution is a procedure performed before surgery. Ogawa et al. [52] determined the change in coagulation and the effect of haemostatic components after haemodilution. Besides T-TAS (AR-chip,) thromboelastometry was also used for the analysis of patients before and after surgery with cardiopulmonary bypass (CPB). The results showed the procoagulatory effect of the combined concentration of the prothrombin complex (PCC) together with fibrinogen. The authors found that after haemodilution, blood flow influenced thrombus formation, and thaemodilution at lower flow rates substantially reduced fibrin deposition. Researchers suggest that blood flow conditions can be used to evaluate the therapeutic efficacy of haemostatic agents [52]. Surgical procedures are divided depending on the specialty and presented in Table 4.

### 3.5. Von Willebrand Factor

This section summarizes five studies related to vWF, most of them (80%) involving patients with vWD. vWD is the most common inherited bleeding disorder caused by a quantitative and/or qualitative abnormality in adhesive plasma protein vWF. [3] In one study, the study group consisted of patients with veno-arterial extracorporeal membrane oxygenation (VA ECMO). In the above-mentioned studies, more than half (60%) used both chips for the analysis (PL-chip and AR-chip). In the study by Ågren et al. [53] only the AR-chip was used, while Mazzeffi et al. [54] conducted a study using only the PL-chip. Nogami et al. [55] and Ågren et al. [53] conducted their studies on patients with a specific type of vWD (type 1 and 3, respectively). Comparison of the studies is presented in Table 5.

Von Willebrand disease is a haemorrhagic diathesis caused by a deficiency or defect of vWF, which is a plasma glycoprotein involved in the process of haemostasis. This disease entity is not uniform; therefore, there are three types of classification (1st, 2nd, 3rd). Type 2 vWD is further subdivided into four subtypes (2A, 2B, 2M, 2N). In the context of the aforementioned vWF, types 1 and 3 are deficient in this factor, while type 2 includes various structural and functional defects [56]. T-TAS can be used in monitoring and diagnosis, as shown by Daidone et al. [57]. In an assessment of the clinical severity of type 1 vWD patients, Nogami et al. [55] found that patients with low vWF ristocetin cofactor activity had a higher blood score than patients with vWF-ristocetin cofactor activity levels and demonstrated that the combined use of vWF-ristocetin cofactor activity and T-TAS analysis could help predict haemorrhagic tendency in vWD type 1. On the other hand, to confirm the usefulness of T-TAS, correlation with parameters that reflect vWF activities, may be necessary with respect to the binding of platelet glycoprotein Ib and binding of collagen. Such analyses are beginning to provide relevant information and may be crucial to elucidating the molecular mechanisms underlying thrombus formation and the reasons for the lack of correlation between PL-T10 and vWF-ristocetin cofactor activity and blood score in some patients [55].

Ågren et al. [53] measured thrombus clot formation under flow conditions by T-TAS, which made it possible to assess the effect of vWF-FVIII concentrate treatment in vWD-3 patients. Results were compared to those obtained by conventional coagulation assays, as well as to results obtained by Multiplate and ROTEM. As expected, administration of the vWF-FVIII concentrate increased the levels of vWF-glycoprotein Ib (GPIb), vWF-antigen (Ag) and FVIII-C in plasma and ristocetin-induced platelet aggregation. Platelet aggregation induced by ADP, TRAP, collagen or arachidonic acid was within reference levels and unaffected by treatment. The defect in platelet-dependent thrombus formation revealed by the T-TAS system may be of clinical importance in vWD-3 [53].

Daidone et al. [57] showed that T-TAS is a useful tool for discriminating and predicting the bleeding score in vWD type 1 patients. vWD patients and healthy subjects were studied with the T-TAS PL and AR microchips developed for in vitro assessment of platelet thrombus formation and fibrin-rich platelet thrombus formation, respectively. According to the authors, T-TAS appeared to be sensitive mainly to the concentration of vWF in plasma and the presence of large multimers. Results showed that blood samples induced no capillary channel occlusion in either chip, meaning that no thrombus was formed. Blood samples were slow to form a thrombus in the PL and/or AR-chip, and blood samples behaved exactly like the blood of normal subjects. These observations suggest that a vWF level above a certain threshold, and a normal vWF multimer pattern, are both needed to guarantee thrombus formation in the T-TAS. To conclude, the authors suggested that T-TAS does not provide an accurate diagnosis of vWD, but points researchers in the right direction and seems to be a useful global preliminary test [57].

Ogiwara et al. [58] studies a small group of patients and suggested that both, PL and AR-chip measurements appear useful for evaluating clinical symptoms of vWD and verifying the effectiveness of the treatment. In the clinical setting, bleeding symptoms as assessment tools and bleeding score are frequently used to diagnose mild to moderate vWD, as well as other rare bleeding disorders, whereas the treatment efficacy with desmopressin and plasma are typically evaluated by monitoring the vWF antigen, the vWF ristocetin cofactor activity and/or subjective clinical changes. Treatment of vWD based on disease severity and the results of minimal laboratory tests represents a more practical approach to disease management. To this end, combined analyses with the specific PL-chip and comprehensive AR-chip assays appear useful for evaluating clinical symptoms of vWD and monitoring treatment efficacy in the clinical setting [58].

Another study on the vWF was conducted by Mazzefii et al. [54] who addressed this topic in the context of patients with extracorporeal oxygenation of the arteriovenous membrane. Platelet function was measured using T-TAS in VA ECMO patients receiving and not receiving vWF concentrate. They observed abnormalities in platelet adhesion and aggregation in the study group, and that administration of vWF concentrates increased adhesion and aggregation but were not normal. Other parameters, such as the level of vWF antigen and the activity of the vWF cofactor ristocetin, were normal or elevated. Significantly low values were recorded for the vWF multimer. The authors suggested the loss or dysfunction of the platelet GP1ba vWF receptor in patients with bleeding complications as an explanation. However, further research is needed to confirm this [54].

### 3.6. Other Uses of T-TAS

Miike et al. [59] used the T-TAS to analyse changes in thrombus formation in blood samples collected from healthy volunteers with no underlying disease. Both platelet aggregation and adhesion were reflected in the time it took for the start of an occlusive thrombus by using the T-TAS system. The reduction in thrombus formation ability changed with the passage of time after hyperbaric oxygen therapy pressurization, and ultimately tended to be similar to that of the control group. As a result, a reduction in the ability to form thrombi was observed, which was presumed to be caused by the effects of pressurization on platelet aggregation and adhesion. In the future, additional tests should be conducted in vivo to further evaluate thrombus formation ability following hyperbaric oxygen therapy, and to elucidate its therapeutic effects [59].

Ono et al. [60] studied the induction of functional platelets from fibroblasts. In this analysis, cultures of mouse and human cells were used, which were subjected to real-time quantitative reverse transcription polymerase chain reaction tests, flow cytometry, morphology, and megakaryocytes infusion. In addition, the PL-chip was used in the T-TAS device to determine the functionality of platelets. It was suggested that the induced megakaryocytes-derived platelets were functional, being incorporated into thrombi under flow conditions [58]. Comparison of these two studies is presented in Table 6.

### 3.7. Summary

The T-TAS system as a new device to assess coagulation and haemostatic function has a completely different principle of measurement from that of conventional POCT devices such as TEG or ROTEM systems. ROTEM and TEG are important methods in the diagnosis and management of treatment in congenital fibrinogen disorders. This method was previously used in a study in patients with hypofibrinogenemia [61]. The TEG and ROTEM systems involve optically measured changes in the mechanical impedance of a sensor pin which generated changes in the elasticity of whole blood in a special cuvette. Both systems are based on the same basic principle of measurement. 

In actual clinical practice, measurement of PT that is commonly used as the indicator of coagulability requires more than 60 min before the result is obtained. These indicators reflect the early stage of the coagulation process. The PT and activated partial thromboplastin coagulability time are influenced by platelets. The activated clotting time (ACT) reflects only the early stage of coagulation, similar to PT and activated partial thromboplastin time (APTT). A review in the Journal of Intensive Care published in 2017 [2] pointed out the principles of measurement by viscoelastic devices and guidelines for the treatment of therapeutic coagulopathy. Whole blood platelet function tests including VerifyNow and Multiplate systems are increasingly utilized for monitoring the efficacy of antiplatelet therapies to prevent coronary arterial thrombosis. However, the assessment of platelet function is limited to adhesion or aggregation in response to a single agonist at a time. Anticoagulation (citrate or hirudin) and a lack of blood flow make platelet aggregometry less physiological. Thromboelastometry (ROTEM or TEG) is frequently used to measure viscoelastic changes due to fibrin formation and platelet activation during thrombus formation in whole blood. There is no “universal test” to assess different antithrombotic agents or combined therapies under physiological conditions. 

T-TAS was initially designed to monitor the effectiveness of antithrombotic agents. The main advantage of the T-TAS system is assessment of clot formation under flow conditions. Therefore, to some extent, T-TAS reflects physiological conditions. A number of studies have reported its potential utility in assessment of antiplatelet drugs, e.g., aspirin, clopidogrel, PAR-1 and PAR-4 antagonists [9,15,22,25,27,28,42] and antithrombotic agents, e.g., direct thrombin and factor Xa inhibitors [10,21,26,40,48,50]. T-TAS has also demonstrated high sensitivity in detecting coagulation disorders such as haemophilia and vWD [32,34,37,53,54,55,56,57,58], as well as platelet function defects for example, such as storage pool disease [6]. Apart from classic studies on the effects of drugs, Skalski et al. [29] and Lis et al. [30] assessed the antiplatelet properties of fractions isolated from plants such as sea buckthorn and dandelion. In both studies it was possible to indicate the anticoagulant potential of selected fractions with the use of T-TAS. In assessing acquired haemorrhagic conditions, T-TAS was able to predict the risk of bleeding in AF patients [27] undergoing catheter ablation. Furthermore, T-TAS has been demonstrated to be a useful tool in the study of thrombus formation in blood taken from animal models such as monkeys [31] and mice [18,33,43]. T-TAS may be useful for patients with diseases such as COVID-19 [7] or PCOS [11]. On the other hand, dabigatran was a therapy in patients in the analysis by Taune et al. [17] In order to determine the optimal concentration for the evaluation of pathologic arterial thrombus formation, there are ongoing evaluations of blood samples from those patients with cerebrovascular disease and coronary atherosclerosis. Summarizing these studies, using T-TAS, it is possible to assess the anticoagulant/antiplatelet effect and determine the differences in haemostasis in individual patients. Regardless of the patient group or the criteria excluding measurements with other devices, T-TAS offers the possibility to assess the potential risk of bleeding or thrombosis.

## 4. Conclusions

The coagulation system involving platelets is a complex mechanism for maintaining haemostasis. Most available studies indicate that T-TAS may be useful for diagnostic issues as a device used to monitor therapy or as a significant tool for predicting bleeding events. However, T-TAS is not only a tool with the potential for diagnostic indications, but by simulating physiological conditions and equipping a camera it enables observation of the effects of drug therapies and defects related to the coagulation system. An additional benefit is the direct observation of the flow and the interactions between blood cells, including the intensity and dynamics of clot formation. T-TAS can be a widely used tool by experienced clinicians; however, it requires more detailed research and analysis. Many studies indicate a number of clinical and prognostic possibilities with the use of T-TAS, but the value of basic research using different groups of patients, observation of flow conditions, clot formation and interactions between blood cells, among other factors, is also of great potential. The device is expected to be of significant value for basic research to observe the interactions and changes within platelets and their microenvironment.

## Figures and Tables

**Table 1:** Comparison of studies with the application of T-TAS in platelet function (AC—anticoagulant, AUC30—area under the flow pressure curve for the first 30 min, COVID-19—coronavirus disease 2019, δ-SPD—platelet δ-storage pool disease, KD—Kawasaki disease, MPV—mean platelet volume, PAI-1—plasminogen activator inhibitor-1, PCOS—polycystic ovary syndrome, POCT—point-of-care, PTF—platelet thrombus formation, ROTEM—thromboelastometry, T1DM—diabetes mellitus type 1, t-PA—tissue plasminogen activator, WT—wild type).

Study	Population	T-TAS Tests	Outcome
Minami, 2015	patients with δ-SPD (*n* = 3), healthy people (*n* = 20)	PL-chip	T-TAS detecting functional disorders of platelets such as δ-SPD
Ghirardello, 2021	patients with COVID-19 (*n* = 61), healthy people (*n* = 32)	PL-chip	The impaired clot formation in COVID-19 patients occurs in the early stages of the disease and correlates with the severity of the disease
Tsujii, 2020	patients with acute KD (*n* = 33)	PL-chip	PTF has an early onset and exhibits poor stability in patients with acute KD
Indemoto, 2017	patients with cardiovascular diseases treated AC (*n* = 78), non-AC (*n* = 25)	PL-chip and AR-chip	T-TAS enables efficient assessment of anticoagulant activity.
Ożegowska, 2020	patients with PCOS (*n* = 39), healthy women (*n* = 11)	AR-chip	PCOS patients have higher AUC30 values
Osiński, 2020	pregnant women with T1DM (*n* = 21), healthy pregnant women (*n* = 15)	AR-chip	MPV, D-dimer and T-TAS measurements can be used to diagnose the prothrombotic state
Yamamoto, 2019	patients with type 2 diabetes (*n* = 10), people without diabetes (*n* = 10)	1. PL-chip and AR-chip 2. PL-chip	T-TAS, determined the reduction of thrombogenicity associated with comprehensive diabetes care and the increase of thrombogenicity associated with hypoglycemia
Hosokawa, 2013	patient with heart disease treated: aspirin (*n* = 20), aspirin and clopidogrel (*n* = 19), healthy people (*n* = 33)	PL-chip	T-TAS in conjunction with conventional platelet function tests and can be used to analyze residual thrombogenicity
Yamaguchi, 2013	healthy people (*n* = 31)	PL-chip and AR-chip	T-TAS can be used to monitor the prevention of thrombotic diseases
Taune, 2017	patients treated with dabigatran (*n* = 30)	AR-chip	T-TAS can be used to detect differences in haemostasis in patients treated with dabigatran
Hosokawa, 2016	healthy people (*n* = 6), mice: WT (*n* = 47) and PAI-1 deficient (*n* = 47)	AR-chip	The arterial shear flow likely influences the anticoagulant efficacy of t-PA
Miike et al., 2021	healthy people (*n* = 10)	PL-chip and AR-chip	Using the POCT methods (T-TAS and ROTEM) it was possible to determine the dose-dependent effect of antithrombin

**Table 2:** Comparison of articles on the topic of monitoring anticoagulant effects (AF—atrial fibrillation, AR10-AUC30—area under the flow pressure curve for the first 30 min for AR-chip tested at flow rate of 10 μL/min, CAD—coronary artery disease, COMPASS—Cardiovascular Outcomes for People Using Anticoagulation Strategies, DAPT—dual antiplatelet therapy, DOACs—direct oral anticoagulants, FXa—activated blood coagulation factor X, NOAC—novel oral anticoagulants, PAF—paroxysmal AF, PEGASUS—Prevention of Cardiovascular Events in Patients with Prior Heart Attack Using Ticagrelor Compared to Placebo on a Background of Aspirin, PL24-AUC10—area under the flow pressure curve for the first 10 min for PL-chip tested at flow rate of 24 μL/min, RFCA—radiofrequency catheter ablation).

Study	Population	T-TAS Tests	Outcome
Hosokawa, 2011	healthy people (*n* = 33)	AR-chip	T-TAS quantifies white thrombus formation
Sugihara, 2016	healthy people (*n* = 20) and patients with AF: treated with rivaroxaban (*n* = 6), apixaban (*n* = 10)	PL-chip and AR-chip	T-TAS is useful for monitoring anticoagulant therapy with FXa inhibitors
Yamazaki, 2016	patients with cerebrovascular diseases treated with antiplatelet therapy (*n* = 94)	PL-chip	T-TAS allows the assessment of platelet inhibition in patients with cerebrovascular disease treated with antiplatelet drugs
Arima, 2015	patients with CAD: not treated with antiplatelet drugs (*n* = 56), treated with aspirin (*n* = 69), aspirin and clopidogrel (*n* = 149)	PL-chip and AR-chip	The T-TAS parameter PL24-AUC10 can be used to evaluate antiplatelet therapy in patients with CAD
Zheng et al., 2021	patients with CAD undergoing PCI treated DAPT with clopidogrel (*n* = 22), prasugrel (*n* = 15), ticagrelor (*n* = 20)	PL-chip	T-TAS can be used in patients treated with antiplatelet drugs to determine primary haemostatic capacity
Hosokawa, 2014	healthy people (*n* = 15)	AR-chip	T-TAS can be used for dose adjustment and selection of the optimal therapy with anticoagulants
Norimatsu, 2014	patient with PAF	PL-chip and AR-chip	T-TAS can be used to evaluate the effect of anticoagulants such as NOAC
Ishii, 2017	patients with AF undergoing RFCA: treated with warfarin (*n* = 29), dabigatran (*n* = 19), rivaroxaban (*n* = 47), apixaban (*n* = 25)	AR-chip	The T-TAS parameter AR10-AUC30 can be used to monitor the effect of anticoagulants such as warfarin and DOACs
Clifford et al., 2021	healthy people (*n* = 24)	PL-chip and AR-chip	The T-TAS technology made it possible to compare the therapies of the PEGASUS and COMPASS in vitro trials with each other
Skalski et al., 2021	healthy people (*n* = 8)	PL-chip	Using the PL-chip (T-TAS), it was possible to demonstrate the anticoagulant potential of four fractions isolated from sea buckthorn
Lis et al., 2021	healthy people (*n* = ?)	PL-chip	T-TAS and flow cytometry enabled the identification of the fraction (C) with the greatest antiplatelet properties
Shimamura et al., 2021	rhesus monkeys (*n* = 4)	PL-chip	The use of T-TAS in this study made it possible to demonstrate the anticoagulant activity of the vaccine in monkeys

**Table 3:** Comparison of studies on the application of T-TAS in bleeding (AF—atrial fibrillation, ARC-HBR—Academic Research Consortium for High Bleeding Risk, AR4-AUC30—area under the flow pressure curve for the first 30 min for AR-chip tested at flow rate of 4 μL/min, AR10-AUC30—area under the flow pressure curve for the first 30 min for AR-chip tested at flow rate of 10 μL/min, CAD—coronary artery disease, CAG—coronarography, CF-LVAD—continuous-flow left ventricular assist device, eGFR—estimated glomerular filtration rate, FVIII—factor VIII, FIX—factor IX, HF—heart failure, NOAC—novel oral anticoagulants, PCI—percutaneous coronary interventions, PFDs—platelet function disorders, PL24-AUC10—area under the flow pressure curve for the first 10 min for PL-chip tested at flow rate of 24 μL/min, vWD -von Willebrand disease).

Study	Population	T-TAS Tests	Outcome
Ogawa et al., 2012	mice FVIII-deficient/wild-type healthy people (*n* = 6)	AR-chip	Observed dense networks of fibrin fibres surrounding platelets in normal blood and coarse fibrin structures with large pores in FIX-inhibited blood.
Nakajima et al., 2020	patients with type 2N vWD (*n* = 5) healthy people (*n* = 20)	PL-chip, AR-chip	The AR-chip enables the prediction of bleeding tendency and the determination of the effectiveness of therapy in patients with type 2N vWD.
Yaoi et al., 2017	patient with type 1 vWD (*n* = 5) healthy people (*n* = 10)	PL-chip	PL-chip enables the identification of impaired haemostasis in the presence of anemia
Takashio et al., 2020	patients with end-stage HF (*n* = 4) patients treated with aspirin and warfarin (*n* = 8)	PL-chip, AR-chip	AR10-AUC30 may apply to stratify the risk of bleeding or thromboembolic disease
Nakajima et al., 2021	patients with VWD (*n* = 22) and PFDs (*n* = 4) healthy people (*n* = 20)	PL-chip, AR-chip	T-TAS may be useful in detecting patients with primary hemostatic disorders
Oimatsu et al., 2017	patients with CAD undergoing PCI (*n* = 313)	PL-chip, AR-chip	PL24-AUC10 may be a marker for perioperative bleeding in patients with CAD undergoing PCI
Ito et al., 2016	patients with AF (*n* = 128)	AR-chip	AR10-AUC30 measured enables the assessment of the efficacy of warfarin and NOAC in patients with AF after catheter ablation
Nakanishi et al., 2021	patients underwent PCI (*n* = 300)	AR-chip	AR10-AUC30 with ARC-HBR allows better prediction of bleeding risk in patients undergoing PCI
Nakanishi et al., 2021	hemodialysis patients undergoing PCI (*n* = 33), patients with eGFR <60 mL/min/1.73 m^2^ undergoing PCI (*n* = 124) and patients with eGFR ≥60 undergoing PCI (*n* = 143)	PL-chip, AR-chip	T-TAS can be used to monitor thrombogenicity to predict the risk of bleeding in hemodlialised patients undergoing PCI
Mitsuse et al., 2020	patients with CAD undergoing CAG or PCI (*n* = 561)	PL-chip, AR-chip	AR10-AUC30 may be useful in predicting 1-year bleeding episodes in CAD patients
Al Ghaithi et al., 2019	patients with coagulation disorders (*n* = 37), healthy people (*n* = 22) and wild-type mice (*n* = 5)	PL-chip, AR-chip	T-TAS can be used to monitor anticoagulant therapy and to test platelet function in patients
Ichikawa et al., 2019	patients with CAD (*n* = 145)	PL-chip, AR-chip	AR4-AUC30 can be used to predict bleeding complications in stable CAD patients receiving oral anticoagulants and antiplatelet agents
Ogawa et al., 2011	patients treated warfarin (*n* = 6), healthy people (*n* = 7)	AR-chip	T-TAS probably illustrates the overall hemostatic activity
Atari et al., 2020	patients who required platelet transfusions (*n* = 10)	PL-chip, AR-chip, HD chip	The new HD chip can determine the risk of bleeding as well as detect recovery of haemostasis after platelet transfusion

**Table 4:** Comparison of studies on the application of T-TAS in surgical procedures (AR10-AUC30—area under the flow pressure curve for the first 30 min for AR-chip tested at flow rate of 10 μL/min, CPB—cardiopulmonary bypass, PL18-AUC10—area under the flow pressure curve for the first 10 min for PL-chip tested at flow rate of 18 μL/min, PPCI—primary percutaneous coronary intervention, TAVI—transcatheter aortic valve implantation, TKA—total knee arthroplasty).

Study	Population	T-TAS Tests	Outcome
Sueta, 2015	patients undergoing TKA (*n* = 20)	PL-chip, AR-chip	The T-TAS parameter AR10-AUC30 can be used to determine the efficacy of the edoxaban
Sueta, 2018	patients undergoing TKA (*n* = 38)	AR-chip	T-TAS parameter–AR10-AUC30 in determining the haemorrhagic risk after TKA surgery
Ishii, 2019	patients undergoing TAVI (*n* = 21)	AR-chip	T-TAS may be useful in analyzing post-TAVI hemorrhagic complications
Kikuchi, 2020	patients undergoing PPCI (*n* = 127)	PL-chip	The T-TAS parameter which is PL18-AUC10 during PPCI reached high values which was associated with impaired reperfusion and large infarct size
Ogawa, 2013	patients before and after CPB (*n* = 15), healthy people (*n* = 12)	AR-chip	The hemodilution procedure causes the blood flow to influence the formation of a thrombus and the subsequent therapy with the use of hemostatic ingredients

**Table 5:** Comparison of studies on the application of T-TAS with respect to the von Willebrand factor (BS—bleeding score calculated with the ISTH Bleeding Assessment Tool, GP1ba—platelet glycoprotein Ib alpha chain, VA ECMO—veno-arterial extracorporeal membrane oxygenation, vWD—von Willebrand disease, vWF—von Willebrand factor).

Study	Population	T-TAS Tests	Outcome
Ågren, 2017	patient with vWD-3 (*n* = 10), healthy people (*n* = 10)	AR-chip	T-TAS revealed abnormalities in thrombus formation that depends on platelets in vWD-3
Mazzeffi, 2019	patients with VA ECMO (*n* = 20), healthy people (*n* = 20)	PL-chip	Reduced platelet adhesion and aggregation in VA ECMO patients may be due to the loss or dysfunction of the vWF GP1ba receptor
Nogami, 2016	patients with vWD-1 (*n* = 50), healthy people (*n* = 30)	PL-chip, AR-chip	T-TAS enables the discrimination and prediction of BS in patients with vWD type 1
Daidone, 2016	patients with vWD (*n* = 30), healthy people (*n* = 20)	PL-chip, AR-chip	T-TAS may be a global pretest for vWD
Ogiwara, 2015	patients with vWD (*n* = 5), healthy people (*n* = 20)	PL-chip, AR-chip	Analysis with PL-chip and AR-chip can be used to assess the clinical symptoms of vWD and to monitor the effectiveness of treatment

**Table 6:** Comparison of studies on the application of atypical use of T-TAS (IMKs—induced MKs, MK—megakaryocyte).

Study	Population	T-TAS Tests	Outcome
Mikke, 2016	healthy people (*n* = 10)	PL-chip	T-TAS made it possible to determine the decrease in thrombus formation capacity in samples subjected to hyperbaric pressure
Ono, 2012	cell cultures	PL-chip	IMKs can be made from fibroblasts, paving the way for further research into the mechanisms of MK differentiation and the production of platelets in this way

## Data Availability

No new data were created or analyzed in this study. Data sharing is not applicable to this article.
